# RNA binding proteins in MLL-rearranged leukemia

**DOI:** 10.1186/s40164-022-00343-5

**Published:** 2022-10-28

**Authors:** Tiffany M. Tran, Dinesh S. Rao

**Affiliations:** 1 Department of Pathology and Laboratory Medicine, David Geffen School of Medicine, UCLA, Los Angeles, CA 90095 USA; 2 Molecular, Cellular, and Integrative Physiology Interdepartmental Ph.D. Program, UCLA, Los Angeles, CA 90095 USA; 3 Jonsson Comprehensive Cancer Center (JCCC), UCLA, Los Angeles, CA 90095 USA; 4 Broad Stem Cell Research Center, UCLA, Los Angeles, CA 90095 USA

**Keywords:** RNA binding protein, MLL, Leukemia

## Abstract

RNA binding proteins (RBPs) have recently emerged as important post-transcriptional gene expression regulators in both normal development and disease. RBPs influence the fate of mRNAs through multiple mechanisms of action such as RNA modifications, alternative splicing, and miR-mediated regulation. This complex and, often, combinatorial regulation by RBPs critically impacts the expression of oncogenic transcripts and, thus, the activation of pathways that drive oncogenesis. Here, we focus on the major features of RBPs, their mechanisms of action, and discuss the current progress in investigating the function of important RBPs in MLL-rearranged leukemia.

## Background

Gene expression can be controlled transcriptionally and post-transcriptionally, and dysregulated gene expression is central in many disease states. Transcriptional regulation of normal development and disease has been extensively studied. Recently, evidence for RNA binding proteins (RBPs) as important post-transcriptional regulators of gene expression has emerged. By virtue of their role in post-transcriptional gene regulation, RBPs are likely to play important roles in development and disease. However, the biological roles and exact mechanisms of action of RBPs in oncogenesis remain to be uncovered. Understanding the complexity and dynamic nature of post-transcriptional gene expression regulation by RBPs in hematologic malignancies has been a major focus of research by several groups in recent years. Amongst the hematologic malignancies, *MLL*-rearranged acute leukemia, including *MLL*-rearranged acute lymphoblastic leukemia (ALL) and acute myeloid leukemia (AML), remain both a significant clinical problem and a pathogenetic enigma. Here, we describe recent advances in understanding the role of RBPs in *MLL*-rearranged leukemia. RBPs have been shown to be aberrantly expressed in *MLL*-rearranged leukemia, with both upregulation and downregulation observed. To our knowledge, there is no current review with a global overview of the pathophysiology of *MLL*-rearranged leukemia and the underlying RBP mechanisms that are intimately connected to its pathogenesis. We believe this is timely and important as RBPs represent novel therapeutic targets in patients with *MLL*-rearranged leukemia, who have a poor prognosis, high risk of relapse, and show resistance to advanced targeted therapies. We begin with a brief introduction to acute leukemia with *MLL* translocations. Next, we discuss the major characteristics of RBPs, including canonical structural features and multiple mechanisms of action that impact gene expression. Lastly, we focus on the current progress of investigating key RBPs in *MLL*-rearranged leukemogenesis and their potential as therapeutic targets.

### MLL-rearranged acute leukemia

Classically, acute leukemia has been thought of as acute lymphoblastic leukemia (ALL) and acute myeloid leukemia (AML), based on morphology and immunophenotype, with ALL further subcategorized as B-ALL and T-ALL. However, we now know that immunophenotypic categorization is insufficient to entirely explain variability in prognosis and therapeutic response, with recurrent chromosomal alterations and mutations playing a highly significant role. Chromosomal rearrangements of the mixed-lineage leukemia (*MLL*, also known as *KMT2A*) gene were originally discovered in mixed phenotype acute leukemia (MPAL, formerly known as mixed-lineage leukemias). It is now recognized that *MLL*-rearranged (*MLL-r*) leukemias comprise approximately 10% of all human leukemias and mostly manifest as B-cell ALL, AML, and acute leukemia of ambiguous lineage [1, 2]. Greater than 70% infant ALL, at least 35% of infant AML, and approximately 10% of adult AML are *MLL-r* [1, 2]. Despite recent advances in therapeutic approaches, patients with *MLL-r* leukemia have very poor outcomes, a high risk of relapse, and show resistance to immune targeted therapies [3, 4]. In *MLL-r* B-ALL, outcomes in both the pediatric and adult populations remain markedly inferior to B-ALL overall [5, 6]. Furthermore, *MLL-r* B-ALL can develop resistance to second line immunotherapeutic approaches, presumably due to lineage plasticity and infidelity [7]. Similarly, *MLL-r* AML is an aggressive subtype of AML, with a poor prognosis and worse overall survival, which may be related to increased rates of relapse [8]. This is thought to be due to the persistence of leukemic stem cells, also known as leukemia-initiating cells (LICs), which evade chemotherapy, have the capability to self-renew, and produce downstream “bulk” leukemia cells [9]. Thus, although significant progress has been made, *MLL-r* leukemias still pose a particular challenge and improved therapeutic approaches are needed.

### Mixed-lineage leukemia 1 (MLL/KMT2A)

*MLL/KMT2A* is the human homolog of the *Drosophila melanogaster* trithorax protein, which is known to regulate embryogenesis and homeotic gene expression (10). Several groups have shown that homozygous deletion of *Mll* in mice is embryonic lethal while heterozygous *Mll* mice display abnormal body patterning and defects in hematopoiesis [11]. *MLL* has been shown to be required for hematopoietic stem cell (HSC) development during both embryonic and adult hematopoiesis [12, 13]. Moreover, *Mll* is required for adult hematopoietic stem and progenitor cell maintenance [13]. The human *MLL* gene is located at the 11q23 locus. The N-terminal portion of MLL has a Menin-binding domain, AT-hook motifs, speckled nuclear localization domains (SNL-1 and SNL-2), and two repression domains (RD1, with a CxxC domain, and RD2) [10, 14, 15]. The center of the MLL protein contains four plant homeodomain (PHD) fingers and a bromodomain while the C-terminal end of MLL contains a SET (Su(var)3–9, enhancer of zeste, trithorax) domain and transcriptional activation domain [10, 16]. The SET domain of MLL is a histone H3 lysine 4 (H3K4) methyltransferase, whose activity may contribute to but may not be necessary for *homeobox (Hox)* gene (*Hoxa9, Hoxa7, Hoxa10*) activation [17, 18], which are known to have important roles in body patterning and hematopoietic development.

### Common MLL fusion partners

**Fig. 1:**
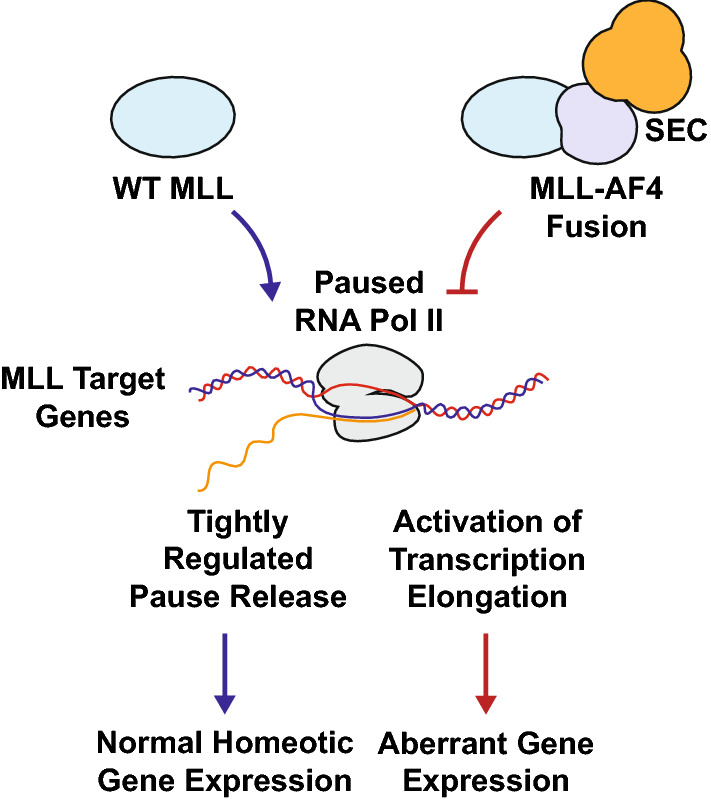
MLL mechanism of transformation. Wild-type (WT) MLL targets important transcriptional regulators of the hematopoietic system. Normal homeotic gene expression depends on the tightly regulated pause and release of RNA Polymerase II (RNA Pol II) on these MLL target genes. Many leukemia associated MLL fusion proteins such as MLL-AF4 recruit the super elongation complex (SEC). This recruitment leads to the premature release of paused RNA Pol II and activation of transcriptional elongation, leading to the aberrant gene expression of MLL target genes that drive leukemogenesis

Wild-type MLL is cleaved into an N-terminal fragment (MLL-N) and a C-terminal fragment (MLL-C) by proteolysis. While MLL-N and MLL-C normally interact in a larger gene regulatory complex, the leukemia associated MLL fusion proteins typically only have the N-terminal portion of MLL and no longer have the capability to interact with MLL-C [19, 20]. The C-terminal and the middle portions of MLL are not usually retained in the majority of MLL fusion proteins. While this loss of interaction would normally lead to the destabilization of MLL-N, in-frame MLL fusion to numerous partner genes likely re-stabilizes the protein [19]. Many MLL fusion partners participate in the recruitment of the super elongation complex (SEC), which includes RNA polymerase II ELL proteins, P-TEFb, and other frequent fusion partner genes including AF4 (AFF1), AF9 (MLLT3), AF10 (MLLT10) and ENL (MLLT1), and the H3K79 histone methyltransferase DOT1L complex [1]. The recruitment of these complexes to MLL fusion protein target genes is thought to result in the enhancement of H3K79 methylation and the upregulation of transcription of these target genes [21, 22]. These targets include important transcriptional regulators in the hematopoietic system, such as those in the *HOX* gene family, thereby amplifying and perpetuating an aberrant, leukemogenic transcriptional gene expression program. This aberrant gene expression dysregulation drives leukemogenesis at the transcriptional level (Fig. 1). However, it should be noted that other MLL fusion partners have been reported, which may entail distinct mechanisms of transformation (e.g., AF6 or AFDN). Here, we discuss the two most common MLL fusion partners: AF4 and AF9.

### MLL-AF4 t(4;11)(q21;q23)

The translocation t(4;11)(q21;q23) of MLL with AF4 results in an in-frame fusion chimeric MLL-AF4 protein. Of more than 90 translocation fusion partner genes, *MLL-AF4* (*KMT2A-AFF1*) is the most common *MLL* fusion protein in patients, occurring in approximately 50% of infant and 75% adult *MLL-r* ALL cases [23]. Clinically, outcomes for *MLL-r* B-ALL patients are poor; specifically, for MLL-AF4 B-ALL the 5-year event-free survival was 13% in the UKALL XI study. A particularly difficult clinical issue is that *MLL-r* B-ALL retains lineage plasticity and infidelity—following treatment with CD19 CAR T-cell therapy or monoclonal antibodies MLL-AF4 ALL can undergo a lineage switch [7]. Relapses with AML, which no longer responds to these surface antigen-targeted treatments, have been reported. Interestingly, in MLL-AF4 leukemia patients, leukemia initiating clones are likely derived from early non-lymphoid committed progenitors, and perhaps a fetal derived cell [24]. However, detailed experimental exploration of such a target cell have been met with limited success, and hence needs further examination.

The *AF4/AFF1* gene is located at the 4q21 locus. In the hematopoietic system, AF4 has shown differential expression in both hematopoietic and nonhematopoietic human cells, with expression particularly high in placental tissues [25]. In mice, deletion of *Af4* resulted in defects in B and T-cell development [26]. AF4 has been shown to interact with proteins involved in the recruitment of the SEC, pTEFb and the histone H3K79 methyltransferase DOT1L, and is a positive regulator of transcriptional elongation [27, 28]. In line with this, *MLL*-*AF4*-driven leukemia is a distinct entity, with a unique gene expression profile showing significant overlap with stem cell programs and enhanced H3K79 methylation at known stem-cell associated genes such as HOXA9, MEIS1, and FLT3 [21, 29].

### MLL-AF9 t(9;11)(p22;q23)

The translocation t(9;11)(p22;q23) results in the MLL-AF9 fusion protein and is predominantly associated with myeloid malignancies. It is the most common MLL fusion protein in AML; accounting for nearly 50% of pediatric *MLL-r* AML and over 25% in adult *MLL-r* AML [23]. MLL-AF9 AML confers an intermediate prognosis in children and adults [8]. However, it should be noted that AML in general has a worse prognosis than B-ALL, and hence the actual prognosis appears to be similar between *MLL-r* AML and *MLL-r* B-ALL [3, 6, 8, 30]. This suggests a common underlying biology leading to similar clinical behavior.

AF9/MLLT3 is located at the 9p22 locus and is a nuclear protein containing sequences associated with transcriptional activator activity. AF9 has extensive homology with another MLL fusion partner gene, ENL, containing a YEATS domain with H1 and H3 acetylation reader capability that plays a critical role in the recruitment of the DOT1L complex for H3K79 methylation and transcriptional elongation [31]. In addition, AF9 and ENL interact with the polycomb repressive complex 1 (PRC1) and the transcriptional repressor BCOR [32, 33]. Similar to *Mll* in embryonic development, mice with homozygous *Af9* deficiency display abnormal body patterning and postnatal lethality [34]. In hematopoiesis, AF9 is a regulator of early erythroid and megakaryocytic cell differentiation [35].

MLL-AF9 AML demonstrates a clear clonal hierarchy of leukemia-initiating cells (LICs) or leukemia stem cells [36]. In murine MLL-AF9 acute myeloid leukemia, these LICs are found at a high frequency and demonstrate expression of mature myeloid lineage-restricted cell markers (CD11b and Gr1) with c-Kit [36]. Like MLL-AF4 ALL, MLL-AF9 AML also displays lineage plasticity and MLL-AF9 LICs display a gene expression profile showing extensive overlap with embryonic stem cell programs. Hence, epigenetic and transcriptional mechanisms for gene expression regulation for these leukemogenic stem cell-related programs are being investigated to exploit for effective targeted therapies.

MLL fusion genes and their downstream effectors have been studied for many years now, resulting in targeted therapies intended to disrupt complexes that promote aberrant transcriptional regulation [22, 27, 37–39]. While these inhibitors have displayed promising results, excess toxicity remains an issue and their therapeutic value is still being evaluated in clinical trials. This highlights the need to further understand mechanisms of leukemogenesis, and post-transcriptional gene regulation by RBPs and other elements of gene regulation is an area of active investigation.

### RBP mechanisms of action

**Fig. 2:**
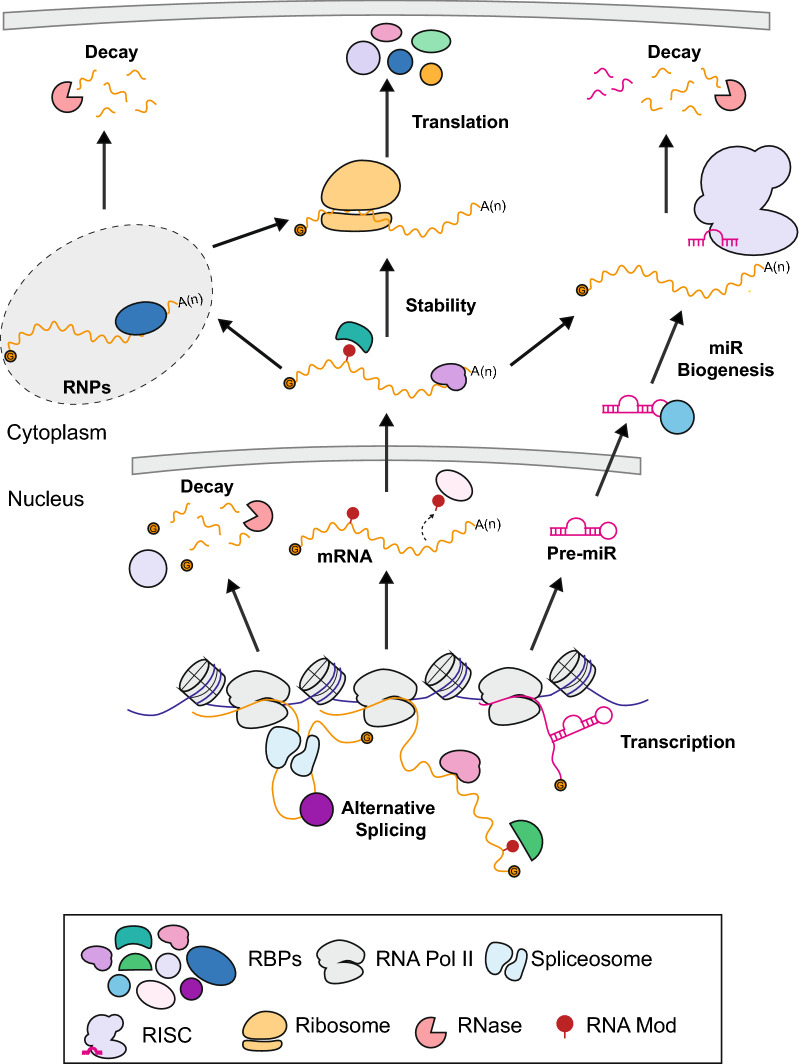
Post-transcriptional gene expression regulation by RBPs. RBPs are responsible for the regulation of the processing and fate of mRNAs. RBPs influence the localization, stability, degradation, and translation of mRNAs through multiple mechanisms of action. RBPs have been shown to have roles in RNA modifications, alternative splicing, the formation of ribonucleoprotein complexes (RNPs), microRNA (miR) mediated regulation of transcripts and miR biogenesis through association with the RNA induced silencing complex (RISC)

Regulation of gene expression can occur at the epigenetic, transcriptional, post-transcriptional, and post-translational levels. For the past 20 years, many groups have made incredible progress in understanding epigenetic and transcriptional gene expression regulation, especially in the context of *MLL-r* leukemia. More recently, post-transcriptional regulation by microRNAs (miRs), long noncoding RNAs (lncRNAs), and RNA binding proteins (RBPs) has been shown to be an equally important component of gene regulation in many developmental and disease processes. Of these, RBPs are, as a class, diverse in their function, and appear to be crucial regulators of the processing and fate of mRNAs (Fig. 2).

### RBP structural features

**Fig. 3:**
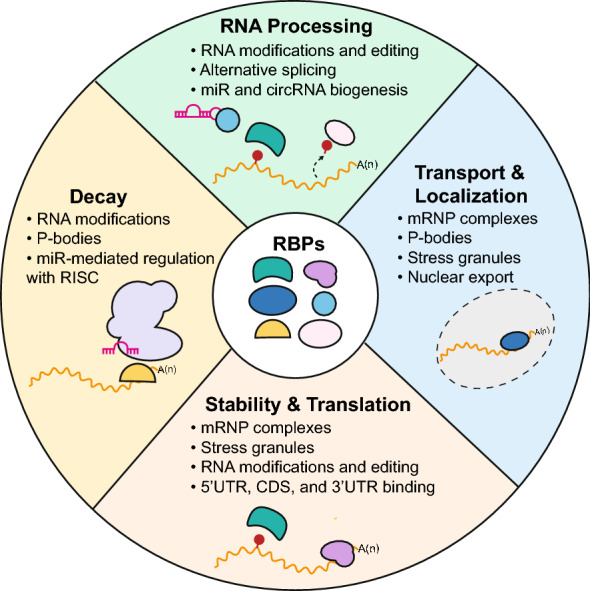
RBP mechanisms of action. The major functions of RBPs include: (1) RNA processing through RNA modifications, RNA editing, alternative splicing, and noncoding RNA biogenesis such as microRNAs (miRs) and circular RNAs (circRNAs). (2) Transport and localization of mRNA through messenger ribonucleoprotein (mRNP) complexes, (P) bodies, and stress granules. (3) Stability and translation of mRNA through mRNP complexes, stress granules, RNA modifications, RNA editing, and binding at the 5′ untranslated region (UTR), coding sequence (CDS), and 3′ UTR. (4) Decay of mRNA through RNA modifications, P-bodies, and miR-mediated regulation and the interaction of the RNA induced silencing complex (RISC). (5) Multiple functions including any of the previously mentioned mechanisms in 1–4. All of these functions critically impact mRNA isoform expression, structure, stability, translation, and decay

RBPs are highly conserved proteins, with expression distributed across a wide array of tissue types. Often, they are thought to have “housekeeping” functions by binding to a myriad of RNA targets [40]. Recent studies with crosslinking immunoprecipitation (CLIP) and RNA sequencing have found RBP binding sites to be distributed across the 3′ untranslated region (UTR), coding sequence (CDS), and 5′ UTR of many RNA targets [41]. The location of these binding sites may further explain their function in gene regulation—for example, the 3′UTR contains many regulatory sequences [42]. RBPs regulate RNA in numerous processes, including transcription, splicing, localization, translation, and degradation, by forming dynamic ribonucleoprotein complexes (RNPs) (Fig. 3). RNPs can have discrete functions based on their composition. So-called ‘classic’ RBPs are characterized by containing one or more RNA binding domains (RBDs) that bind to specific RNA sequences and structural motifs. The most well-defined and prevalent RBDs are the RNA recognition motif (RRM), hnRNP K homology (KH), DEAD/DEAH helicase, and zinc-finger domains [43].

Despite their expression in a wide array of tissues and housekeeping roles, many RBPs have been shown to cause tissue-specific effects. Underlying this is the idea that RBPs regulate particular sets of mRNAs as cell-type specific “regulons” [42, 44]. This specificity may be explained by the following combination of features: (i) RNA targets of RBPs have cell-type-specific expression; (ii) regulatory RNP complexes depend on the stoichiometry of RBPs, specific interacting proteins and coding/non-coding RNAs; and (iii) post-translational modification of proteins, e.g., signaling networks, that can influence the formation of RNP complexes [45]. In addition to active translational machinery and the spliceosome, which have been reviewed elsewhere, RBP action can be related to their roles in processing (P) bodies and stress granules, as well as in microRNA-mediated and epitranscriptomic regulation.

### Post-transcriptional gene expression regulation by RBPs

**Fig. 4:**
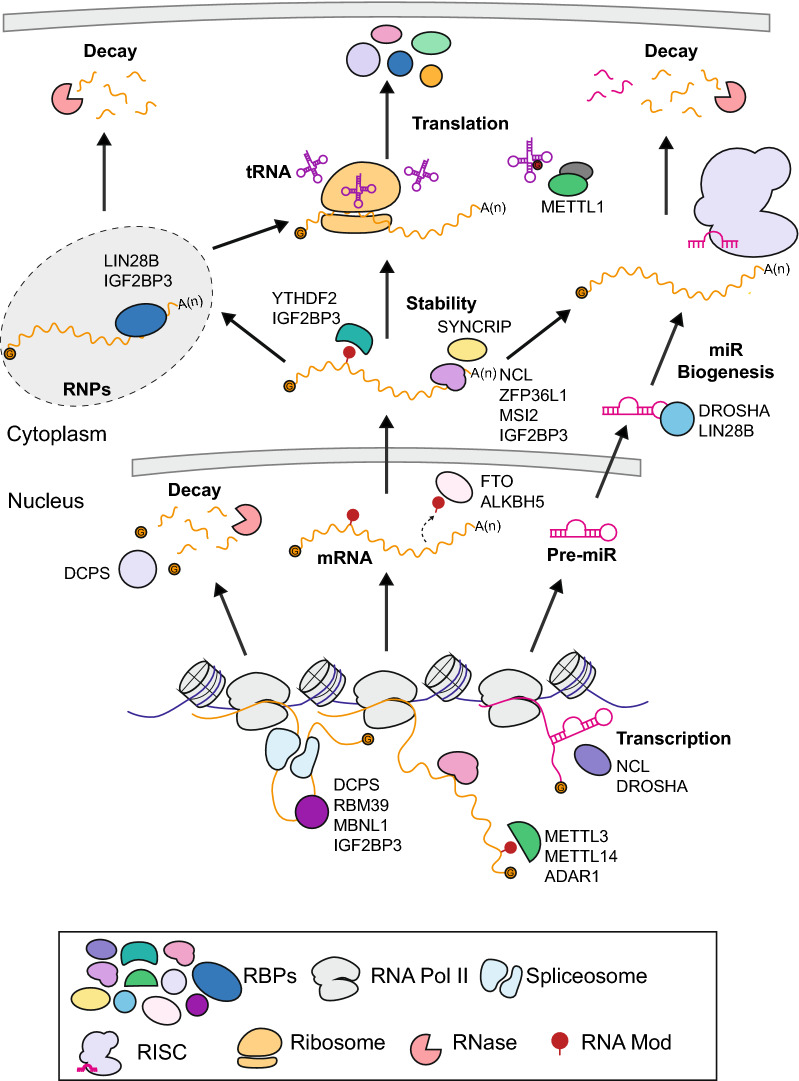
Post-transcriptional gene expression regulation by RBPs directly assessed in *MLL-r* leukemia. METTL3 and METTL14 function as m^6^A writers, FTO and ALKBH5 function as m^6^A erasers, and YTHDF2 functions as an m^6^A reader. ADAR1 catalyzes A-to-I RNA editing. RBPs involved in alternative splicing include the trans-acting splicing factor RBM39, the 5′ cap binding enzyme DCPS, and MBNL1. NCL has been shown to bind to the 3′UTR of mRNA transcripts and be required for miR biogenesis. ZFP36L1 binds to the 3′UTR of mRNA transcripts such as CDK6. DROSHA has been implicated to be recruited by MLL-AF4 and MLL-AF9 to target genes encoding miRs as well as function in the cytoplasm in non-canonical miR biogenesis. The METTL1/WDR4 heterodimeric complex catalyzes m^7^G modifications on tRNA. Multifunctional RBPs: LIN28B localizes in P-bodies, stress granules, and mRNP complexes and has an important function in miR biogenesis. MSI2 has been shown to bind to the 3′UTR of mRNA transcripts and interact with SYNCRIP to target the same transcripts. MSI2 may also have a function in alternative splicing. IGF2BP3 has been shown to function in alternative splicing, RNA modifications as an m^6^A reader, localization within ribonucleoprotein complexes (RNPs) and stress granules, and binding to the 3′ untranslated region (3′UTR) of mRNA transcripts impacting their association with the RNA induced silencing complex (RISC) to regulate the stability, translation, and degradation of target transcripts

Emerging evidence has shown RBPs to be important post-transcriptional regulators that can drive oncogenesis. The best understood class of RBPs, splicing factors, which are recurrently mutated in many types of hematologic malignancy, have been reviewed elsewhere [46]. Importantly, recurrently mutated splicing factors are less common in in *MLL-*r acute leukemia [47]. Rather, dysregulation of non-splice factor RBPs have been associated with poor clinical outcomes and may be markers of disease aggressiveness. Many of these studies have determined that the high expression and aberrant activity of RBPs has a critical role in driving leukemia progression and aggressiveness through the regulation of alternatively spliced, modified, and stabilized mRNA leukemogenic transcripts. However, the biological role and exact mechanism of action of many RBPs in leukemogenesis remains to be uncovered, particularly in appropriate in vivo models. Understanding the complex and dynamic post-transcriptional gene expression regulation by RBPs will give insight to potential targeted therapies. Here, we will examine these mechanisms with examples of RBPs that have been specifically characterized in *MLL-r* leukemia (Fig. 4; Table 1).

**Table 1:** Characterized RBPs in MLL-AF9 and MLL-AF4 Leukemia

RBP	MLL FP	Type of leukemia	Mechanism	Function	Characterization	Refs.
METTL3	MLL-AF9	AML	RNA modification	m6A writer	Human primary AML cells, Human MOLM-13 cell line, MLL-AF9 Flt3-ITD mouse model, CRISPR dropout screen	[51, 52]
METTL14	MLL-AF9	AML	RNA modification	m6A writer	Human primary AML cells, Human MM6 cell line, MLL-AF9 Mettl14 CKO mouse model	[53]
FTO	MLL-AF9	AML	RNA modification	m6A eraser	Human primary AML cells, Human MONOMAC-6 and MV4-11 cell lines, MLL-AF9 Fto knockdown and heterzygous knockout mouse model	[54]
ALKBH5	MLL-AF9, MLL-AF4	AML	RNA modification	m6A eraser	Human primary AML cells, Human MONOMAC-6, NOMO1 MOLM-13, THP-1, MV4-11 cell lines, MLL-AF9 Alkbh5 CKO mouse model	[55, 56]
YTHDF2	MLL-AF9	AML	RNA modification	m6A reader	Human primary AML cells, Human THP-1 cell line, Ythdf2 CKO Hoxa9 Meis1 mouse model	[57]
METTL1	MLL-AF9	AML	RNA modification	m7G writer	Human primary AML cells, Human MOLM-13 and THP-1 cell lines, MLL-AF9/Flt3^ITD/+^ deletion mouse model, MLL-AF9 AML xenograft mouse model	[63]
ADAR1	MLL-AF9	AML	RNA editing	A-to-I editing	Human primary AML cells, Human THP-1 cell line, MLL-AF9 Adar1 CKO mouse model	[72, 73]
RBM39	MLL-AF9	AML	Alternative splicing	Splicing factor	RBD CRISPR/Cas9 screen, Human primary AML cells, Human MOLM-13 and THP-1 cell lines, MLL-AF9 NrasG12D mouse model	[81]
DCPS	MLL-AF9, MLL-AF4	AML	Alternative splicing	5′ cap binding enzyme	Human MOLM-13, THP-1, and MV4-11 cell lines, Genome-wide CRISPR/Cas9 screen using MLL/AF9-Cas9 mouse model, PDX AML models	[84]
MBNL1	MLL-AF9, MLL-AF4	ALL, AML	Alternative splicing	Splicing, mRNA decay	Human primary MLL-r leukemia cells, Human THP-1, RS4;11, MOLM13, SEM and MV4-11 cell lines, MLL-Af4 mouse model, MLL-AF9 PDX with Mbnl1 knockdown mouse model, MLL-AF9 Mbnl1 KO mouse model	[86, 87]
DROSHA	MLL-AF9, MLL-AF4	AML, ALL	Primary miR processing	miR biogenesis	Human primary AML cells, Human SEMK2, PER377, MV4-11 cell line, Mll^PTD/wt^/Flt3^ITD/ITD^ mouse model	[95, 96]
NCL	MLL-AF9, MLL-AF4	AML	mRNA stability and 3′UTR association	Ribosome biogenesis, miR biogenesis	Human primary AML and ALL cells, Human MOLM-13 and MV4-11 cell lines, Cell-line MLL-AF9 xenograft NCL knockdown mouse model	[108, 112, 113]
ZFP36L1	MLL-AF9	AML	mRNA stability and 3′UTR association	MZ B cell maintenance, thymopoiesis	Human primary MLL-r AML cells, Human THP-1 cell line	[114]
LIN28B	MLL-AF9	AML	Multiple mechanisms of action	Embryonic stem cell pluripotency, self-renewal, fetal lymphopoiesis, miR biogenesis	Human MLL-r AML cells, Human THP-1 cell line, AML xenograft mouse model, MLL-AF9 AML mouse model	[120, 121]
MSI2	MLL-AF9	AML	Multiple mechanisms of action	Self-renewal and pluripotency of embryonic stem cells, regulation of HSCs	Human primary AML cells, Human NOMO-1 and THP-1 cell lines, MLL-AF9 AML Msi2 conditional knockout mouse model	[135, 136, 137, 139]
SYNCRIP	MLL-AF9	AML	Multiple mechanisms of action	Regulation of transcripts involved in myeloid leukemia stem cells	Human primary AML cells, Human NOMO-1, MOLM-13, and THP-1 cell lines, MLL-AF9 leukemia in vivo shRNA screen, MLL-AF9 AML Syncrip KO mouse	[138]
IGF2BP3	MLL-AF4	ALL	Multiple mechanisms of action	Cell migration, survival, differentiation and stem cell renewal	Human primary ALL cells, Human RS4;11 and SEM cell lines, IGF2BP3 enforced expression mouse model, MLL-Af4 Igf2bp3 KO leukemia model	[143, 58]

### RNA modifications

Until quite recently, most known RNA modifications were mainly identified on transfer RNA (tRNA) and ribosomal RNA (rRNA). Now, numerous groups have shown that RNA modifications occur on mRNA as well as non-coding RNA and can have profound impacts on gene expression. These RNA modifications include but are not limited to N6-methyladenosine (m^6^A), 5-methylcytosine (m^5^C), N1-methyladenosine (m^1^A), 7-Methylguanosine (m^7^G) capping, pseudouridine, and adenosine-to-inosine (A-to-I) editing [48]. All these modifications can critically change the secondary structure and folding of RNA and, thus, its interactions with other RNA and proteins. These RNA modifications have been excellently reviewed elsewhere by many groups and, thus, will be briefly discussed here. As the most directly characterized mRNA modification in *MLL-r* leukemia, we will focus the majority of our discussion on the m^6^A modification and its RNA modifiers.

### m^6^A

The most prevalent eukaryotic mRNA modification is methylation of adenosine at position 6, m^6^A, which has been a recent major interest in cancer. The deposition of m^6^A is catalyzed by so-called m^6^A writers. The central m^6^A writer complex consists of the main catalytic methyltransferase-like 3 (METTL3) subunit, METTL14, which recognizes the substrate as the RNA binding scaffold, and other cofactors including WTAP and RBM15. Notably, the deposition of m6A can occur as a co-transcriptional event guided by histone H3 trimethylation at lysine 36 (H3K36me3), a transcriptional elongation marker, which is recognized and bound by METTL14 [49]. This results in the recruitment of the remaining components of the m^6^A writer complex to mediate the deposition of m^6^A. The m^6^A modified mRNAs are then recognized by m^6^A readers, such as the YTHDF, hnRNP and IGF2BP family of RBPs, which regulate the localization, stability, and translation of these mRNAs. Finally, the m^6^A modification is removed by m^6^A erasers, FTO and ALKBH5 [50].

### m^6^A writers

Of the proteins involved in RNA modification, the m^6^A METTL3-METTL14 writer complex, m^6^A reader YTHDF2, and m^6^A eraser FTO and ALKBH5 have all been shown to play a role in *MLL-r* leukemia. AML cells show high expression of METTL3 compared to other cancer types and human cord blood CD34 + cells [51, 52]. Through a CRISPR dropout screen on murine Cas9 hematopoietic stem and progenitor cells (HSPCs) with enforced expression of MLL-AF9 and *Flt3* internal tandem duplication (ITD), METTL3 was identified to be required for the growth of AML cells. METTL3 deficiency led to cell cycle arrest, leukemic cell differentiation, and the inability to initiate leukemia in immunodeficient mice [51]. Supporting this, shRNA-mediated knockdown of METTL3 in the MLL-AF9 expressing human AML cell line, MOLM-13, led to apoptosis, differentiation, and a delay in leukemia development in recipient mice [52]. Bone marrow mononuclear cells from primary AML patients showed the highest expression of METTL14 in *MLL-r* samples, METTL14 expression was induced by MLL-AF9 expression, and depletion led to apoptosis, leukemic cell differentiation, and a diminution of in vivo leukemogenesis using both shRNA and conditional genetic murine models [53]. Mechanistically, these studies attribute the function of METTL3 in MLL-AF9 AML to be due to the deposition of m^6^A on known oncogenic targets such as SP1, MYC, MYB, and BCL2 resulting in translation promotion [51–53].

### m^6^A erasers

FTO and ALKBH5 have both been demonstrated to be highly expressed in *MLL-r* AML patient samples [54–56]. In an MLL-AF9 mouse model, *Fto* deficiency resulted in a significant delay in leukemogenesis due to the regulation of its targets, ASB2 and RARA, by decreasing the m^6^A levels of these transcripts [54]. ALKBH5 expression has been determined to be significantly overexpressed in both MLL-AF9 and MLL-AF4 AML cell lines and to be correlated with poor prognosis [55, 56]. In MLL-AF9 transformed *Alkbh5* deficient HSPCs, *Alkbh5* was found to be required for leukemogenesis as well as the development and self-renewal capacity of LICs [55]. In parallel, a significant delay in leukemogenesis was observed in an *Alkbh5* conditional knockout MLL-AF9 model [56]. The authors also found that *Alkbh5* is required for LIC maintenance and the ability to reconstitute MLL-AF9 secondary transplanted mice [56]. Furthermore, in human MLL-AF9 and MLL-AF4 translocated AML cell lines, shRNA-mediated knockdown of *Alkbh5* significantly increased the survival of xenograft mice and the latency of AML [55]. The authors attribute the oncogenic function of ALKBH5 to be from the targeting of and regulating the mRNA stability of TACC3 and AXL, respectively [55, 56].

### m^6^A readers

Akin to METTL3 and METTL14, the m^6^A reader YTHDF2 was also found to be highly expressed in MLL-AF9 AML cells compared to control cells. In a conditional *Ythdf2* mouse model, deletion of *Ythdf2* significantly increased the latency of disease, decreased the number of LICs and decreased the ability of these LICs to reconstitute mice [57]. YTHDF2 was found to target m^6^A modified mRNAs, such as *Tnfrsf1b* for decay [57]. In addition, we recently implicated the m^6^A reader IGF2BP3 in MLL-Af4 driven acute leukemia in a murine model [58], although it is not yet clear if the preferential mechanism is via a modulation of m^6^A modified transcripts.

### m^7^G

A second mRNA modification, namely 7-methylguanosine (m^7^G) modification, occurs at the initial stages of transcription and is distributed at the 5′ cap region of mRNA and internally within tRNA and rRNA [59]. The m^7^G modification on tRNA is catalyzed by the METTL1/WDR4 heterodimeric complex and is recognized by eukaryotic translation initiation factor 4E (eIF4E), which is indispensable for cap-dependent translation initiation [60, 61]. eIF4E has been shown to be overexpressed in many human tumors and to contribute to cancer progression, tumorigenesis, and metastasis [62]. In AML patient samples, METTL1 and WDR4 expression are elevated at both the mRNA and protein level [63]. Furthermore, METTL1 was shown to be differentially expressed in an MLL-AF9/Flt3^ITD/+^ primary murine AML model. Deletion of METTL1 in this model led to the inhibition of AML cell growth and significantly reduced colony formation of LICs [63]. In an MLL-AF9 AML xenograft mouse model, METTL1-knockout led to an increase in overall survival and reduction in tumor burden. Enforced expression of METTL1 in the human MLL-AF9 expressing cell lines MOLM-13 and THP-1 led to an increase in proliferation. The underlying mechanism was observed to be from an increase in m^7^G modifications on tRNA mediated by the METTL1/WDR4 complex leading to the reduction of ribosome pause efficacy on mRNAs enriched with AGA codons, influencing the translation of transcripts important to cell cycle progression [63]. Lastly, when comparing m^7^G methylation peaks of mRNA in AML cells with drug-resistant AML cells, one recent study found a significant difference in the level and distribution of m^7^G methylation [64]. This suggests that m^7^G methylation may have an important role in the regulation of drug resistance in AML, but the functional implications on specific methylated leukemogenic genes remains to be determined.

### A-to-I editing

A-to-I editing occurs by site-selective editing of adenosine to inosine mediated by the family of adenosine deaminases acting on RNA (ADAR) enzymes, which are double-stranded RNA binding proteins that include ADAR1, ADAR2, and ADAR3 [65, 66]. It is the most common type of RNA editing in mammals, with millions of editing sites detected in humans due to the improvement of accurate computational detection and technical high-throughput sequencing methods. ADAR1 has been best characterized in chronic myeloid leukemia (CML), where it has been shown to promote the malignant reprogramming of myeloid progenitors to LICs, LIC self-renewal, and the propagation of malignant progenitors [67–69]. Elevated expression of ADAR1 is seen in pediatric B-ALL and adult AML, although expression within specific subtypes, such as in *MLL-r* leukemia is unclear [70, 71]. In the human MLL-AF9 AML cell line THP-1, differentiation by phorbol-myristate acetate led to an increase in expression of ADAR1 and ADAR2, with a corresponding increase in A-to-I editing [72]. Lastly, in a murine MLL-AF9 model, tamoxifen induced ADAR1 deletion led to a significant increase in survival of the mice and increase in apoptosis of the MLL-AF9 HSPCs [73]. With proposed roles in LIC self-renewal and maintenance as well as the likely combinatorial regulatory mechanisms with miRs and m^6^A writers on target transcripts, further investigation on the role of ADARs specifically in *MLL-r* leukemia is needed to understand the functional importance and implications of A-to-I editing by ADARs in the context of this disease.

### Other RNA modifications: m^5^C, m^1^A and pseudouridine

Other RNA modifications exist and have been described in other physiological and pathological contexts, but not in *MLL-r* leukemia, to our knowledge. The m^5^C modification occurs by the methylation of carbon 5 in cytosine in RNA, which is catalyzed by the NOL1/NOP2/SUN (NSUN) methyltransferases family or DNMT2 [74]. In AML cells resistant to azacitidine, a DNA hypomethylating agent, it was determined that m^5^C modifications along with NSUNs and DNMT2 were increased [75]. m^1^A modifications occur on the nitrogen in the first position on adenine. Found in tRNA, rRNA, and, to a lesser extent compared to m^6^A, in mRNA, it has been found to be enriched near the start codon upstream of the first splice site [76]. Pseudouridine was the first RNA modification discovered and, overall, the most abundant RNA modification, particularly in rRNA and tRNA. While it is found in mRNA to a lesser extent, this modification has been shown to impact translation rate and accuracy [77]. The significance of m^5^C, m^1^A, and pseudouridine modifications in post-transcriptional gene regulation in *MLL-r* leukemia remains to be determined.

### Alternative splicing

Alternative splicing results in multiple mRNA isoforms from one gene and is a fundamental process of gene expression regulation. In cancer, cells frequently express aberrantly spliced isoforms that provide a proliferative or survival advantage [78]. A large RNP complex, known as the spliceosome, is responsible for splicing and includes the subunits U1, U2, U4, U5, and U6 small nuclear RNPs along with several other factors, described below [79]. Splice site (ss) recognition is regulated by both cis-acting splicing regulatory elements, such as enhancers and silencers, and trans-acting splicing factors such as the RBPs SF3B1, U2AF1, RBM39, and SRSF2. These splicing factors mediate the recruitment and positioning of spliceosome components to pre-mRNA and are commonly mutated in numerous cancer types, including leukemia [80]. Although mutation of splicing factors is relatively uncommon in *MLL-r* acute leukemia, alternative splicing changes appear to be widespread, as in other leukemia subtypes [46, 80]. Alternative splicing can result in several different changes to the mature mRNA molecule. Of these, intron retention has recently been reported to be a mechanism of transcriptome diversification and tumor-suppressor gene inactivation in cancer and, specifically, in leukemia [81, 82].

### RBPs involved in alternative splicing

A recent publication highlighted the importance of this mechanism in *MLL-r* acute leukemia. RBM39 is a splicing factor that is known to interact with SF3B1 and U2AF65 splicing factors [83]. In the MLL-AF9 Nras^G12D^ mouse model, sgRNAs against the RRM domains of *Rbm39,* initially identified in a CRISPR screen, led to a significant increase in leukemia latency [81]. Furthermore, they found that deletion of RBM39 led to an increase in apoptosis and decrease in the growth of human MLL-AF9 AML cell lines. Mechanistically, RBM39 was shown to target known HOXA9 targets and promote intron retention. Furthermore, the authors utilized the small molecule inhibitor, indisulam (E7070), which selectively degrades RBM39, on human MLL-AF9 AML cell lines and observed dose-dependent decreases in the expression of RBM39 and HOXA9 targets as well as increased apoptosis. In vivo, indisulam treatment of AML transplanted mice resulted in a significant decrease in leukemia burden. Interestingly and perhaps not surprisingly, the authors observed preferential sensitivity of human AML cell lines with spliceosomal mutations to sulfonamides.

Through a genome-wide CRISPR/Cas9 screen in MLL-AF9 primary murine cells, DCPS, a mRNA 5′ cap binding enzyme shown to have a role in mRNA decay, was identified to be significantly depleted and critical for AML survival [84]. Furthermore, the authors found that DCPS interacts with pre-mRNA processing machinery including spliceosomes and Nucleosome Remodeling Deacetylase (NuRD) subunits. In addition, they determined that treatment with the DCPS inhibitor, RG3039, resulted in decreased proliferation in human *MLL-r* cell lines and PDX models, as well as increased apoptosis and increased differentiation. RNA-seq on treated cell lines revealed pre-mRNA mis-splicing from DCPS depletion.

MBNL1 is an RNA binding protein that has been characterized to have a role in regulating alternative splicing and mediating mRNA decay [85]. Furthermore, it has been shown to be a direct MLL-AF4 target in human ALL cell lines and a murine MLL-Af4 model [86, 87]. *Mbnl1* deficiency in MLL-AF9 transformed murine HSPCs significantly increased the survival and the latency of disease of transplanted mice [87]. In addition, a MBNL1-specific inhibitor showed selective activity in human MLL-AF9 and MLL-AF4 AML cell lines, albeit at high concentrations. The authors showed that MBNL1 regulates alternative splicing, mostly associated with intron exclusion, in known leukemogenic genes such as DOT1L and SETD1A.

With the prevalence of alternative splicing mutations and the critical regulatory functions of these splicing factors on leukemogenic genes, this aspect of gene expression regulation seems to be promising for novel therapeutic strategies. The results from these studies utilizing specific inhibitors are encouraging. More clinical evaluation is needed, however, to assess the true therapeutic value of these inhibitors.

### P-bodies and stress granules

In eukaryotic cells, mRNAs undergoing active translation are generally protected from degradation, and the two processes—translation and degradation—generally are in competition with each other. mRNAs can be thought of existing in a translating and a non-translating pool. It was discovered that the non-translating mRNA pool can be sequestered in the cytoplasm in two types of granules: P-bodies and stress granules, which additionally are composed of specific RBPs. P-bodies are dynamic, cytoplasmic RNP complexes that are highly dependent on the pool of non-translating mRNAs present for assembly. P-bodies contain RBPs involved in mRNA decay and translation repression, as well as components of the nonsense-mediated decay pathway (NMD), and components of the miRNA machinery [88]. Stress granules and P-bodies may have common constituents, but stress granules also contain translation initiation factors such as the eIF4F complex of RBP proteins including eIF4E, poly-A binding proteins (PABP), and the 40S ribosomal subunit as well as other translational regulatory factors. While stress granules can be induced during stress conditions, they also appear during other cellular states in which translation initiation is stopped [88]. *MLL-r* leukemia was recently shown to have fewer P-bodies than non-*MLL-r* leukemia [89], suggesting that *MLL-r* leukemia has a smaller pool of non-translating mRNAs. More generally, post-transcriptional gene regulation depends on RBPs, miRNAs and possibly other non-coding RNA molecules, although it is not clear if these interactions occur in specific cytoplasmic compartments, such as the P-body or stress granule.

### mRNA stability and 3′UTR-binding RBPs

The 3′UTR of mRNA has been shown to play a critical role in regulating transcript stability and translation. Perhaps the best elucidated mechanism is that of microRNAs (miRs), which are small non-coding RNAs that bind to target sequences in the 3′UTR of mRNAs. Primary miRs (pri-miRs) undergo processing by a microprocessor complex that contains the ribonuclease Drosha and several other factors including DiGeorge critical region 8 (DGCR8) in the nucleus [90]. These shortened hairpin structures known as pre-miRs are then translocated to the cytoplasm and cleaved by another type III RNAse, Dicer, to produce mature miRs [91]. Mature miRs form a ribonucleoprotein complex, known as RISC, with Argonaute (Ago) proteins, Dicer, and the HIV transactivating response RNA-binding protein (TRBP), which influence the fate of mRNAs through mRNA cleavage or translational repression [92–94]. While miRs themselves have been shown to be aberrantly expressed in numerous cancers, including hematologic malignancies, the investigation into the dysregulation of these major proteins within the miR biosynthetic pathway is limited. Interestingly, Drosha has been shown to be recruited by MLL-AF4 and MLL-AF9 fusion proteins to target genes encoding miRs [95]. DROSHA has also been shown to localize within the cytoplasm to mediate the maturation of miR-155, a BIC-155 long noncoding (lnc) RNA-hosted oncogenic miRNA, in FLT3-ITD AML leukemic blasts, a Mll^PTD/wt^/Flt3^ITD/ITD^ mouse model, and MV4-11 cells [96]. Notably, Ago2 contains nuclease activity and has been shown to have a key role in slicer-independent miR biogenesis in B lymphoid and erythroid cell development and function [97, 98]. In AML, Ago2 was shown to modulate the gene expression program that drives human monocytic cell fate determination [99]. Ago-RNA complexes have regulatory roles in transcription, splicing, and genome maintenance [100].

Binding sites for miRs are mostly located in the 3′UTR, which also contains regulatory elements bound by RBPs, such as AU-rich elements (AREs) recognized by ARE RBPs like HuR/ELAVL1 and AUF1 [42]. Although RBPs can bind to different motifs within the 3′UTR of a miR target, many RBPs often compete to bind to the same motif within the 3′UTR. Taken with the finding that miRs and mRNAs have been shown to differentially and preferentially bind to Ago proteins in a cell context-specific manner in AML, this highlights a multifaceted regulatory network [101]. This combinatorial regulation can lead to often highly complex patterns—RBPs have been shown to have cooperative interactions with miRs and, conversely, to act as “safe houses” for target mRNAs against miR-mediated decay [102–104]. Similarly, combinatorial interactions occur at other cis-regulatory sites within the 3′UTR, such as AU-rich elements (ARE), where a host of competitive and cooperative RBP interactions can occur [42].

Perhaps the one of the most characterized RBPs is HuR, which belongs to the ELAV family of proteins. In the hematopoietic system, HuR has been shown to be essential for hematopoietic progenitor cell maintenance and the B-cell antibody response [105, 106]. Although the function of HuR has not been directly assessed in *MLL-r* leukemia, it has been shown to regulate and associate with other ARE RBPs that have been shown to be aberrantly expressed in *MLL-r* leukemia such as EIF4E, NCL, and ZFP36L1 [107, 108]. Furthermore, numerous studies have shown HuR to have a critical functional role in regulating the fate of important leukemogenic transcripts, such as BCL-2 and MYC, through its interactions with the RISC complex to modulate miR-mediated repression [109, 110]. HuR can function to both cooperate with miRs, such as the case with let-7 in the promotion of miR-mediated repression of MYC, and to compete with miRs to protect the mRNA from decay, such as with NCL [110, 111].

NCL, nucleolin, has numerous cellular functions including regulation of RNA polymerase I transcription, processing of pre-ribosomal RNA and ribosome assembly, and nucleo-cytoplasmic transport. NCL binds to an ARE in the antiapoptotic Bcl-2 3′UTR and protects the transcript from exosomal decay from another ARE RBP, AUF1, suggesting that NCL and AUF1 compete to bind Bcl-2 in opposing roles in regulating its stability [108]. In MLL-AF9 AML, NCL is required for the proper processing of the pri-miRNA precursor of miR-15a/16 and its expression is directly correlated to the expression of miR-15a/16 [112]. Finally, high NCL expression correlated with DNA methyltransferase (DNMT) upregulation and shorter survival in AML patients. In functional experiments, the authors found that NCL1 played an oncogenic role in AML and was inversely correlated with DNMT expression [113]. Inhibition of NCL with the anti-nucleolin aptamer AS1411 led to a significant decrease in colony formation and DNA hypomethylation in MV4-11 cells.

ZFP36L1 is an ARE RBP that belongs to the zinc finger protein homolog 36 family. It is important in the regulation of various lymphoid subsets, including marginal zone B-cells and in thymopoiesis. In *MLL-r* AML patients, ZFP36L1 was significantly downregulated compared to normal control samples [114]. Furthermore, shRNA-mediated knockdown of ZFP36L1 in THP-1 cells and CD34 + HSPCs significantly impaired monocytic and macrophage differentiation. Mechanistically, ZFP36L1 directly targeted the 3′UTR of CDK6, a cell cycle regulator required in *MLL-r* AML and downregulated its expression [114].

The above studies show a role for several RBPs targeting cis-regulatory elements within the 3′UTR of oncogenic and tumor suppressive mRNA transcripts. In addition, competition and cooperative interactions occur in binding, with downstream impacts on gene expression. It is likely these combinatorial regulatory complexes are critically important in perpetuating an oncogenic gene expression program and may be exploited for potential targeted therapies. While combinatorial post-transcriptional regulation by RBPs and miRs have been shown in a range of cancer types, less is known about these mechanisms in *MLL-r* leukemia. An important area for further work remains to elucidate the basis of the cis-regulatory elements—sequence as well as structural elements and covalent modifications in the target mRNA—is an important area for further work.

### LIN28 RBPs: impacts on miR biogenesis

In addition to the convergence of post-transcriptional gene regulation on regulatory elements in the 3′UTR, recent work has identified that specific RBPs play important roles in miR biogenesis. miRNAs are encoded in cellular genes, and the primary transcript undergoes processing by endoribonucleases DROSHA, DGCR8 and DICER, amongst others. The processing steps are regulated by several factors [115], amongst which the LIN28 RBPs feature prominently. The LIN28 family of RBPs consists of two paralogs, LIN28A and LIN28B. LIN28A and LIN28B are upregulated in approximately 15% of human tumors and cancer cell lines, with activation associated with poor prognosis and advanced malignancy [116]. They both have been shown to have important roles in numerous developmental processes including embryonic stem cell pluripotency and self-renewal as well as fetal lymphopoiesis [117, 118]. LIN28B is an oncofetal RBP, with high expression during early embryogenesis, low expression in differentiation and adult tissues, and over-expression in cancer cells [116]. In fetal lymphopoiesis, *Lin28b* and *Igf2bp3*, another oncofetal RBP discussed below, directly interacted and together mediate the fetal–adult hematopoietic switch in B lymphopoiesis through the activation of the fetal transcription program [119].

LIN28 has been shown to be multifunctional: it localizes in P-bodies and stress granules and associates with polysomes and mRNP complexes. However, a large body of evidence points to its role in regulating miR biogenesis: LIN28 binds to let-7 miR precursors, inhibiting Dicer processing and thereby regulating miR-mediated gene expression repression [117–119]. In the human MLL-AF9 AML cell line THP-1, LIN28B knockdown resulted in a significant reduction in tumor burden in an AML xenograft model, resulting from de-repression of let-7, which in turn regulated another RBP, IGF2BP1 [120]. In a further interconnection between LIN28 and miR function in *MLL-r* AML, miR-150 processing was also controlled by LIN28 [121]. This resulted in downregulation of miR-150 in patient AML samples and in murine experimental models, and in turn, de-repression of miR-150 targets MYB and FLT3. Importantly, restoring miR-150 expression inhibited the growth of MLL-AF9 leukemia. Hence, inhibiting this leukemogenic pathway of miRNA processing became a priority for several groups, with the eventual identification of a small molecule inhibitor of LIN28 [122], with others reported subsequently. This is an exciting development, given that RBPs were traditionally thought to be “undruggable”. Nonetheless, further investigation is needed in testing these inhibitors specifically in *MLL-r* leukemia, and in defining the specificity of regulated miR biogenesis.

### Circular RNAs

RBPs have also been shown to bind to some circular RNAs (circRNAs), which are generated by back-splicing of pre-mRNA transcripts, in a cell-type specific manner [123, 124]. Briefly, it has been shown that circRNAs have impacts on gene expression regulation by their potential functions as miR sponges, as competing endogenous RNAs for both miRs and RBPs, and in a cis-regulatory role for transcription [125–127]. The interaction with RBPs form distinct circRNA-protein complexes (circ-RNPs), such as with IGF2BP3 [128]. These interactions have been shown to be critical for circRNA biogenesis, regulated by RBPs such as ADAR1, MBL, and QKI, and cellular processes such as cell cycle progression with the circ-Foxo3–p21–CDK2 ternary complex [125, 129–131]. Transcribed exons from chimeric genes from chromosomal translocations, such as MLL-AF4 and MLL-AF9, can lead to aberrant fusion-circRNAs (f-circRNAs), such as circ4 and f-circM9, respectively [132, 133]. Both f-circRNAs have been shown to contribute to leukemogenesis and required for leukemic cell survival. In FLT3-ITD AML, circMYBL2 was found to regulate FLT3 translation through the recruitment of the RBP PTBP1 to bind its mRNA, thereby promoting disease progression [134]. More work is needed to functionally assess and understand the clinical implications of the molecular interactions between circRNAs and RBPs in leukemogenesis, especially in *MLL-r* subtypes, in which both circRNAs and RBPs are aberrantly expressed.

### RBPs with other mechanisms of action

Here, we will discuss RBPs in *MLL-r* leukemia that show multiple roles in RNA homeostasis, including those in RNA modifications, alternative splicing, and miR-mediated regulation/RISC association. The Musashi family of RBPs, including MSI1 and MSI2, have been shown to play critical roles in *MLL-r* leukemia. MSI2, in particular, is required for the self-renewal and pluripotency of embryonic stem cells, regulates the hematopoietic stem cell compartment, and is overexpressed in AML where it is associated with poor survival [135, 136]. Utilizing an MLL-AF9 AML *Msi2* conditional knockout model, Michael Kharas’ group determined that *Msi2* deficiency led to a significant delay in leukemia development, decrease in leukemic cell infiltration, and a significant decrease in LICs (CD11b + Kit +) [137]. The authors attributed this striking phenotype to the function of MSI2 in maintaining the LIC self-renewal gene expression program by targeting leukemogenic MLL target transcripts *Hoxa9*, *Myc*, and *Ikzf2.* The Kharas group subsequently determined that depletion of SYNCRIP, a novel MSI2 interacting protein resulted in increased apoptosis, decreased proliferation, and increased differentiation in human MLL-AF9 AML cell lines and inhibited leukemia progression in an in vivo murine model [138]. Similar to MSI2, SYNCRIP was required for leukemia progression by utilizing an MLL-AF9 AML *Syncrip* knockout mouse model [138]. Mechanistically, SYNCRIP appeared to act on the same transcripts as MSI2 and led to the stabilization of translation of selected targets such as HOXA9. The exact molecular mechanism of translational repression by MSI2/SYNCRIP remains elusive; as MSI2 has been shown to bind to the 3′UTR of mRNAs but may also have some role in regulating alternative splicing. In a promising development, a small molecule inhibitor of MSI2, Ro 08–2750, was identified by the same group and shows anti-leukemic activity in vitro and downstream impacts on gene expression [139].

The Insulin like growth factor 2 mRNA binding protein (IGF2BP) family consists of three functionally and structurally related paralogs: IGF2BP1, IGF2BP2, and IGF2BP3. The IGF2BPs have been implicated in having numerous critical cellular functions including cell migration, survival, differentiation, and stem cell renewal [119, 140]. IGF2BP1 and IGF2BP3 have both been characterized as oncofetal RBPs that are highly expressed during embryogenesis, lowly expressed in healthy adult tissues, and strongly re-expressed in malignant tissues [140]. Both of these RBPs contain classic RBDs including four KH domains and two RRM domains [140]. Mechanistically, both have been shown to have multiple mechanisms of action on the localization, stability, and translation of mRNA transcripts including direct regulation in mRNPs and stress granules, miR-mediated regulation, in RNA modifications as m^6^A readers, and, potentially, in alternative splicing (particularly for IGF2BP3) [104, 141, 142] (Fig. 4).

IGF2BP1 has been shown to have an important role in tumorigenesis in many different cancers and correlated with a poor prognosis. Multiple studies, including our own, have determined that IGF2BP1 is specifically highly expressed in ETV-RUNX1 translocated B-ALL and lowly expressed in *MLL-r* B-ALL patient samples [143–145]. Here, we will focus on IGF2BP3, which is specifically overexpressed in *MLL-r* leukemia.

In the hematopoietic system, immunohistochemical studies demonstrate that IGF2BP3 is highly expressed in various mature B-cell neoplasms and differential regulation of this protein has been observed in B-ALL [144, 146]. As in other cancer types, IGF2BP3 overexpression was found to be associated with aggressive behavior in B-ALL. Mechanistically, akin to IGF2BP1, IGF2BP3 protects let-7 target transcripts, including HMGA2 and LIN28B, by disrupting RISC association and upregulating expression in development and cancer [102, 104]. With its recently reported function as an m^6^A reader, many studies have sought to understand how RNA modifications in turn regulate the mechanism of IGF2BP3 in both development and cancer. Interestingly, while binding to m^6^A RNA has been established in a range of cancer types, the contribution of IGF2BP3 to the stability of these transcripts has not been fully elucidated. IGF2BP3 appears to stabilize m^6^A modified transcripts by binding to them, akin to other readers such as YTHDF1-3.

Our group identified IGF2BP3 to be specifically overexpressed in *MLL-r* B-ALL patient samples and is an important regulator of gene expression in *MLL*-r B-ALL [143]. We determined that enforced expression of IGF2BP3 in the bone marrow of mice leads to a pathologic expansion of HSPCs, in a manner dependent on RNA binding. Mechanistically, we determined that IGF2BP3 interacts primarily with the 3′UTR of its target leukemogenic transcripts, such as MYC and CDK6, resulting in an upregulation of transcript and protein [143]. Furthermore, IGF2BP3 binding to CDK6 and MYC led to pathologic HSPC expansion in vivo.

We further explicitly tested the requirement for *Igf2bp3* in a bona-fide in vivo model of MLL-Af4 driven leukemogenesis [58, 86]. Utilizing an *Ig2bp3* knockout MLL-Af4 driven leukemia mouse model, we determined that *Igf2bp3* deficiency significantly increased the survival of MLL-Af4 transplanted mice and decreased the numbers and self-renewal capacity of MLL-Af4 LICs. Interestingly, we determined that IGF2BP3 binding sites were not only in the 3′UTR as previously discovered but also in intronic regions and 5′ and 3′ ss of its target transcripts. We found that IGF2BP3 targets and modulates the expression of MLL target transcripts within the *Hoxa* locus as well as components of the Ras signaling pathway, both key regulators of leukemogenesis, through multiple post-transcriptional mechanisms including alternative splicing. Together, our findings have shown IGF2BP3 is a critical post-transcriptional gene expression regulator of MLL-AF4 mediated leukemogenesis [58].

## Conclusions and future directions

In summary, RBPs have been shown to be important post-transcriptional gene expression regulators in both normal development and cancer. Mechanistically, they are a diverse group of proteins, acting in different cellular compartments, at different stages of the gene expression paradigm, and in the regulation of specific transcripts. Using high-throughput sequencing methods, a great deal of progress has been made in improving our understanding of which transcripts are bound and where in the transcript the binding event happens. However, the cis-regulatory basis of mRNA binding appears to be more complex than a simple linear mRNA sequence; cooperative binding of multiple short sequences with spacing rules and structural constraints are likely the rule for many of these RNA binding proteins. Technologies that can profile RNA secondary structure, such as SHAPE-seq and others, may help us better understand the spatial relationships between the target mRNA molecules and the bound RBPs [147, 148]. Additionally, the development of native RNA sequencing technologies may help us better define chemical modifications on specific nucleotides in combination with immunoprecipitation techniques to define the specificity of RNP complexes [149].

While infrequently mutated, the aberrant expression of RBPs is highly associated with disease aggressiveness, poor prognosis, therapy resistance, and relapse in *MLL-r* leukemia. Although the mechanisms are highly complex, the convergence of regulation by several RBPs onto common pathways suggests their central importance. Many groups have made progress in identifying small molecule inhibitors for these RBPs, some of which were discussed here, but toxicity remains an issue, given that many RBPs are ubiquitously expressed in normal tissues and perform globally important functions. However, oncofetal RBPs such as LIN28B and IGF2BP3 allow specific targeting in *MLL-r* leukemia cells and, thus, could be particularly valuable therapeutically. Interestingly, the differential expression of the IGF2BP family of RBPs highlight the potential for a common post-transcriptional, oncogenic function by different members of the same family of RBPs in unique molecular subtypes of disease. Further investigation into the RBP families mentioned in this review will help further elucidate such specific post-transcriptional functions, which may be leveraged in the future as therapeutic strategies. Furthermore, combinatorial therapeutic approaches—adding RBP inhibition to upstream, MLL-targeted transcriptional inhibition or to downstream CDK4/6 inhibition—may yield more efficacious approaches. Hence, the careful and detailed study of pathogenetic mechanisms of post-transcriptional regulation by RBPs in *MLL-r* leukemia will yield new and important therapeutic options in this difficult-to-treat disease.

## Data Availability

Not applicable.

## References

[1] Krivtsov AV, Armstrong SA (2007). MLL translocations, histone modifications and leukaemia stem-cell development. Nat Rev Cancer.

[2] Muntean AG, Hess JL (2012). The pathogenesis of mixed-lineage leukemia. Annu Rev Pathol.

[3] Moorman AV, Ensor HM, Richards SM, Chilton L, Schwab C, Kinsey SE (2010). Prognostic effect of chromosomal abnormalities in childhood B-cell precursor acute lymphoblastic leukaemia: results from the UK Medical Research Council ALL97/99 randomised trial. Lancet Oncol.

[4] Pui C-H, Carroll WL, Meshinchi S, Arceci RJ (2011). Biology, risk stratification, and therapy of pediatric acute leukemias: an update. J Clin Oncol.

[5] Piciocchi A, Messina M, Elia L, Vitale A, Soddu S, Testi AM (2021). Prognostic impact of KMT2A-AFF1-positivity in 926 BCR-ABL1-negative B-lineage acute lymphoblastic leukemia patients treated in GIMEMA clinical trials since 1996. Am J Hematol.

[6] Hunger SP, Mullighan CG (2015). Acute lymphoblastic leukemia in children. N Engl J Med.

[7] Gardner R, Wu D, Cherian S, Fang M, Hanafi LA, Finney O (2016). Acquisition of a CD19-negative myeloid phenotype allows immune escape of MLL-rearranged B-ALL from CD19 CAR-T-cell therapy. Blood.

[8] Döhner H, Weisdorf DJ, Bloomfield CD (2015). Acute myeloid leukemia. N Engl J Med.

[9] Hope KJ, Jin L, Dick JE (2004). Acute myeloid leukemia originates from a hierarchy of leukemic stem cell classes that differ in self-renewal capacity. Nat Immunol.

[10] Tkachuk DC, Kohler S, Cleary ML (1992). Involvement of a homolog of Drosophila trithorax by 11q23 chromosomal translocations in acute leukemias. Cell.

[11] Yu BD, Hess JL, Horning SE, Brown GAJ, Korsmeyer SJ (1995). Altered Hox expression and segmental identity in Mll-mutant mice. Nature.

[12] Ernst P, Fisher JK, Avery W, Wade S, Foy D, Korsmeyer SJ (2004). Definitive hematopoiesis requires the mixed-lineage leukemia gene. Dev Cell.

[13] Jude CD, Climer L, Xu D, Artinger E, Fisher JK, Ernst P (2007). Unique and independent roles for MLL in adult hematopoietic stem cells and progenitors. Cell Stem Cell.

[14] Yokoyama A, Cleary ML (2008). Menin critically links MLL proteins with LEDGF on cancer-associated target genes. Cancer Cell.

[15] Xia Z-B, Anderson M, Diaz MO, Zeleznik-Le NJ (2003). MLL repression domain interacts with histone deacetylases, the polycomb group proteins HPC2 and BMI-1, and the corepressor C-terminal-binding protein. Proc Natl Acad Sci.

[16] Zeleznik-Le NJ, Harden AM, Rowley JD (1994). 11q23 translocations split the "AT-hook" cruciform DNA-binding region and the transcriptional repression domain from the activation domain of the mixed-lineage leukemia (MLL) gene. Proc Natl Acad Sci USA.

[17] Mishra Bibhu P, Zaffuto Kristin M, Artinger Erika L, Org T, Mikkola Hanna KA, Cheng C (2014). The histone methyltransferase activity of MLL1 is dispensable for hematopoiesis and leukemogenesis. Cell Rep.

[18] Terranova R, Agherbi H, Boned A, Meresse S, Djabali M (2006). Histone and DNA methylation defects at Hox genes in mice expressing a SET domain-truncated form of Mll. Proc Natl Acad Sci.

[19] Hsieh JJD, Ernst P, Erdjument-Bromage H, Tempst P, Korsmeyer SJ (2003). Proteolytic cleavage of MLL generates a complex of N- and C-terminal fragments that confers protein stability and subnuclear localization. Mol Cell Biol.

[20] Yokoyama A, Kitabayashi I, Ayton PM, Cleary ML, Ohki M (2002). Leukemia proto-oncoprotein MLL is proteolytically processed into 2 fragments with opposite transcriptional properties. Blood.

[21] Krivtsov AV, Feng Z, Lemieux ME, Faber J, Vempati S, Sinha AU (2008). H3K79 methylation profiles define murine and human MLL-AF4 leukemias. Cancer Cell.

[22] Yokoyama A, Lin M, Naresh A, Kitabayashi I, Cleary ML (2010). A higher-order complex containing AF4 and ENL family proteins with P-TEFb facilitates oncogenic and physiologic MLL-dependent transcription. Cancer Cell.

[23] Meyer C, Burmeister T, Gröger D, Tsaur G, Fechina L, Renneville A (2018). The MLL recombinome of acute leukemias in 2017. Leukemia.

[24] Bardini M, Woll PS, Corral L, Luc S, Wittmann L, Ma Z (2015). Clonal variegation and dynamic competition of leukemia-initiating cells in infant acute lymphoblastic leukemia with MLL rearrangement. Leukemia.

[25] Chen C-S, Hilden JM, Frestedt J, Domer PH, Moore R, Korsmeyer SJ (1993). The chromosome 4q21 gene (AF-4/FEL) is widely expressed in normal tissues and shows breakpoint diversity in t(4;11)(q21;q23) acute leukemia. Blood.

[26] Isnard P, Coré N, Naquet P, Djabali M (2000). Altered lymphoid development in mice deficient for the mAF4 proto-oncogene. Blood.

[27] Bitoun E, Oliver PL, Davies KE (2007). The mixed-lineage leukemia fusion partner AF4 stimulates RNA polymerase II transcriptional elongation and mediates coordinated chromatin remodeling. Hum Mol Genet.

[28] Mueller D, Bach C, Zeisig D, Garcia-Cuellar M-P, Monroe S, Sreekumar A (2007). A role for the MLL fusion partner ENL in transcriptional elongation and chromatin modification. Blood.

[29] Guenther MG, Lawton LN, Rozovskaia T, Frampton GM, Levine SS, Volkert TL (2008). Aberrant chromatin at genes encoding stem cell regulators in human mixed-lineage leukemia. Genes Dev.

[30] Mrózek K, Marcucci G, Nicolet D, Maharry KS, Becker H, Whitman SP (2012). Prognostic significance of the European LeukemiaNet standardized system for reporting cytogenetic and molecular alterations in adults with acute myeloid leukemia. J Clin Oncol.

[31] Li Y, Wen H, Xi Y, Tanaka K, Wang H, Peng D (2014). AF9 YEATS domain links histone acetylation to DOT1L-mediated H3K79 methylation. Cell.

[32] Hemenway CS, de Erkenez AC, Gould GCD (2001). The polycomb protein MPc3 interacts with AF9, an MLL fusion partner in t(9;11)(p22;q23) acute leukemias. Oncogene.

[33] Srinivasan RS, Erkenez ACD, Hemenway CS (2003). The mixed lineage leukemia fusion partner AF9 binds specific isoforms of the BCL-6 corepressor. Oncogene.

[34] Collins EC, Appert A, Ariza-McNaughton L, Pannell R, Yamada Y, Rabbitts TH (2002). Mouse Af9 is a controller of embryo patterning, like Mll, whose human homologue fuses with Af9 after chromosomal translocation in leukemia. Mol Cell Biol.

[35] Pina C, May G, Soneji S, Hong D, Enver T (2008). MLLT3 regulates early human erythroid and megakaryocytic cell fate. Cell Stem Cell.

[36] Somervaille TCP, Cleary ML (2006). Identification and characterization of leukemia stem cells in murine MLL-AF9 acute myeloid leukemia. Cancer Cell.

[37] Koss C, Nance S, Connelly M, Ma J, Shelat A, Cotton A (2014). Targeted inhibition of the MLL transcriptional complex by proteosome inhibitors elicits a high response rate in relapsed/refractory MLL rearranged leukemia. Blood.

[38] Daigle SR, Olhava EJ, Therkelsen CA, Basavapathruni A, Jin L, Boriack-Sjodin PA (2013). Potent inhibition of DOT1L as treatment of MLL-fusion leukemia. Blood.

[39] Grembecka J, He S, Shi A, Purohit T, Muntean AG, Sorenson RJ (2012). Menin-MLL inhibitors reverse oncogenic activity of MLL fusion proteins in leukemia. Nat Chem Biol.

[40] Gerstberger S, Hafner M, Tuschl T (2014). A census of human RNA-binding proteins. Nat Rev Genet.

[41] Singh G, Pratt G, Yeo GW, Moore MJ (2015). The clothes make the mRNA: past and present trends in mRNP fashion. Annu Rev Biochem.

[42] Mayr C (2016). Evolution and biological roles of alternative 3'UTRs. Trends Cell Biol.

[43] Gebauer F, Schwarzl T, Valcárcel J, Hentze MW (2021). RNA-binding proteins in human genetic disease. Nat Rev Genet.

[44] Keene JD, Lager PJ (2005). Post-transcriptional operons and regulons co-ordinating gene expression. Chromosome Res.

[45] Iadevaia V, Gerber AP (2015). Combinatorial control of mRNA Fates by RNA-binding proteins and non-coding RNAs. Biomolecules.

[46] Chen S, Benbarche S, Abdel-Wahab O (2021). Splicing factor mutations in hematologic malignancies. Blood.

[47] Lavallée V-P, Baccelli I, Krosl J, Wilhelm B, Barabé F, Gendron P (2015). The transcriptomic landscape and directed chemical interrogation of MLL-rearranged acute myeloid leukemias. Nat Genet.

[48] Barbieri I, Kouzarides T (2020). Role of RNA modifications in cancer. Nat Rev Cancer.

[49] Huang H, Weng H, Zhou K, Wu T, Zhao BS, Sun M (2019). Histone H3 trimethylation at lysine 36 guides m(6)A RNA modification co-transcriptionally. Nature.

[50] Vu LP, Cheng Y, Kharas MG (2019). The Biology of m_6_A RNA methylation in normal and malignant hematopoiesis. Cancer Discov.

[51] Barbieri I, Tzelepis K, Pandolfini L, Shi J, Millán-Zambrano G, Robson SC (2017). Promoter-bound METTL3 maintains myeloid leukaemia by m6A-dependent translation control. Nature.

[52] Vu LP, Pickering BF, Cheng Y, Zaccara S, Nguyen D, Minuesa G (2017). The N6-methyladenosine (m6A)-forming enzyme METTL3 controls myeloid differentiation of normal hematopoietic and leukemia cells. Nat Med.

[53] Weng H, Huang H, Wu H, Qin X, Zhao BS, Dong L (2018). METTL14 inhibits hematopoietic stem/progenitor differentiation and promotes leukemogenesis via mRNA m(6)A modification. Cell Stem Cell.

[54] Li Z, Weng H, Su R, Weng X, Zuo Z, Li C (2017). FTO plays an oncogenic role in acute myeloid leukemia as a N(6)-methyladenosine RNA demethylase. Cancer Cell.

[55] Shen C, Sheng Y, Zhu AC, Robinson S, Jiang X, Dong L (2020). RNA demethylase ALKBH5 selectively promotes tumorigenesis and cancer stem cell self-renewal in acute myeloid leukemia. Cell Stem Cell.

[56] Wang J, Li Y, Wang P, Han G, Zhang T, Chang J (2020). Leukemogenic chromatin alterations promote AML leukemia stem cells via a KDM4C-ALKBH5-AXL signaling axis. Cell Stem Cell.

[57] Paris J, Morgan M, Campos J, Spencer GJ, Shmakova A, Ivanova I (2019). Targeting the RNA m(6)A reader YTHDF2 selectively compromises cancer stem cells in acute myeloid leukemia. Cell Stem Cell.

[58] Tran TM, Philipp J, Bassi JS, Nibber N, Draper JM, Lin TL (2022). The RNA-binding protein IGF2BP3 is critical for MLL-AF4-mediated leukemogenesis. Leukemia.

[59] Zhang L-S, Liu C, Ma H, Dai Q, Sun H-L, Luo G (2019). Transcriptome-wide mapping of internal N_7_-methylguanosine methylome in mammalian mRNA. Mol Cell.

[60] Lin S, Liu Q, Lelyveld VS, Choe J, Szostak JW, Gregory RI (2018). Mettl1/Wdr4-mediated m_7_G tRNA methylome is required for normal mRNA translation and embryonic stem cell self-renewal and differentiation. Mol Cell.

[61] Borman AM, Michel YM, Malnou CE, Kean KM (2002). Free poly(A) stimulates capped mRNA translation in vitro through the eIF4G-Poly(A)-binding protein interaction*. J Biol Chem.

[62] De Benedetti A, Graff JR (2004). eIF-4E expression and its role in malignancies and metastases. Oncogene.

[63] Orellana EA, Liu Q, Yankova E, Pirouz M, De Braekeleer E, Zhang W (2021). METTL1-mediated m_7_G modification of Arg-TCT tRNA drives oncogenic transformation. Mol Cell.

[64] Zhang B, Li D, Wang R. Transcriptome profiling of N7-methylguanosine modification of messenger RNA in drug-resistant acute myeloid leukemia. Front Oncol. 2022;12. 10.3389/fonc.2022.926296 PMC9294171 35865472

[65] Bass BL, Nishikura K, Keller W, Seeburg PH, Emeson RB, O'Connell MA (1997). A standardized nomenclature for adenosine deaminases that act on RNA. RNA.

[66] Levanon EY, Eisenberg E, Yelin R, Nemzer S, Hallegger M, Shemesh R (2004). Systematic identification of abundant A-to-I editing sites in the human transcriptome. Nat Biotechnol.

[67] Jiang Q, Crews LA, Barrett CL, Chun H-J, Court AC, Isquith JM (2013). ADAR1 promotes malignant progenitor reprogramming in chronic myeloid leukemia. Proc Natl Acad Sci.

[68] Jiang Q, Isquith J, Zipeto MA, Diep RH, Pham J, Delos Santos N (2019). Hyper-editing of cell-cycle regulatory and tumor suppressor RNA promotes malignant progenitor propagation. Cancer Cell.

[69] Zipeto Maria A, Court Angela C, Sadarangani A, DelosSantosNathaniel P, Balaian L, Chun H-J (2016). ADAR1 activation drives leukemia stem cell self-renewal by impairing Let-7 biogenesis. Cell Stem Cell.

[70] Ma C-H, Chong J-H, Guo Y, Zeng H-M, Liu S-Y, Xu L-L (2011). Abnormal expression of ADAR1 isoforms in Chinese pediatric acute leukemias. Biochem Biophys Res Commun.

[71] Xiao H, Cheng Q, Wu X, Tang Y, Liu J, Li X (2019). ADAR1 may be involved in the proliferation of acute myeloid leukemia cells via regulation of the Wnt pathway. Cancer Manag Res.

[72] Rossetti C, Picardi E, Ye M, Camilli G, D'Erchia AM, Cucina L (2017). RNA editing signature during myeloid leukemia cell differentiation. Leukemia.

[73] Peng L, Yang X, Zhang Y, Hu T, Wang W, Wang X (2015). Effect of ADAR1 on the development of MLL-AF9 induced murine AML. Zhonghua Xue Ye Xue Za Zhi.

[74] Trixl L, Lusser A (2019). The dynamic RNA modification 5-methylcytosine and its emerging role as an epitranscriptomic mark. WIREs RNA.

[75] Cheng JX, Chen L, Li Y, Cloe A, Yue M, Wei J (2018). RNA cytosine methylation and methyltransferases mediate chromatin organization and 5-azacytidine response and resistance in leukaemia. Nat Commun.

[76] Dominissini D, Nachtergaele S, Moshitch-Moshkovitz S, Peer E, Kol N, Ben-Haim MS (2016). The dynamic N(1)-methyladenosine methylome in eukaryotic messenger RNA. Nature.

[77] Li X, Ma S, Yi C (2016). Pseudouridine: the fifth RNA nucleotide with renewed interests. Curr Opin Chem Biol.

[78] Obeng EA, Stewart C, Abdel-Wahab O (2019). Altered RNA processing in cancer pathogenesis and therapy. Cancer Discov.

[79] Matera AG, Wang Z (2014). A day in the life of the spliceosome. Nat Rev Mol Cell Biol.

[80] Taylor J, Lee SC (2019). Mutations in spliceosome genes and therapeutic opportunities in myeloid malignancies. Genes Chromosomes Cancer.

[81] Wang E, Lu SX, Pastore A, Chen X, Imig J, Chun-Wei Lee S (2019). Targeting an RNA-binding protein network in acute myeloid leukemia. Cancer Cell.

[82] Jung H, Lee D, Lee J, Park D, Kim YJ, Park W-Y (2015). Intron retention is a widespread mechanism of tumor-suppressor inactivation. Nat Genet.

[83] Loerch S, Maucuer A, Manceau V, Green MR, Kielkopf CL (2014). Cancer-relevant splicing factor CAPERα engages the essential splicing factor SF3b155 in a specific ternary complex. J Biol Chem.

[84] Yamauchi T, Masuda T, Canver MC, Seiler M, Semba Y, Shboul M (2018). Genome-wide CRISPR-Cas9 screen identifies leukemia-specific dependence on a Pre-mRNA metabolic pathway regulated by DCPS. Cancer Cell.

[85] Ho TH, Charlet-B N, Poulos MG, Singh G, Swanson MS, Cooper TA (2004). Muscleblind proteins regulate alternative splicing. EMBO J.

[86] Liu W, Cai H, Lin M, Zhu L, Gao L, Zhong R (2016). MicroRNA-107 prevents amyloid-beta induced blood-brain barrier disruption and endothelial cell dysfunction by targeting Endophilin-1. Exp Cell Res.

[87] Itskovich SS, Gurunathan A, Clark J, Burwinkel M, Wunderlich M, Berger MR (2020). MBNL1 regulates essential alternative RNA splicing patterns in MLL-rearranged leukemia. Nat Commun..

[88] Decker CJ, Parker R (2012). P-bodies and stress granules: possible roles in the control of translation and mRNA degradation. Cold Spring Harb Perspect Biol..

[89] Zhu S, Cheng X, Wang R, Tan Y, Ge M, Li D (2020). Restoration of microRNA function impairs MYC-dependent maintenance of MLL leukemia. Leukemia.

[90] Gregory RI, Yan KP, Amuthan G, Chendrimada T, Doratotaj B, Cooch N (2004). The microprocessor complex mediates the genesis of microRNAs. Nature.

[91] Kim VN, Han J, Siomi MC (2009). Biogenesis of small RNAs in animals. Nat Rev Mol Cell Biol.

[92] Bartel DP (2004). MicroRNAs: genomics, biogenesis, mechanism, and function. Cell.

[93] Bartel DP (2009). MicroRNAs: target recognition and regulatory functions. Cell.

[94] Kuchenbauer F, Mah SM, Heuser M, McPherson A, Rüschmann J, Rouhi A (2011). Comprehensive analysis of mammalian miRNA* species and their role in myeloid cells. Blood.

[95] Nakamura T, Canaani E, Croce CM (2007). Oncogenic All1 fusion proteins target Drosha-mediated microRNA processing. Proc Natl Acad Sci.

[96] Nguyen LXT, Zhang B, Hoang DH, Zhao D, Wang H, Wu H (2021). Cytoplasmic DROSHA and non-canonical mechanisms of MiR-155 biogenesis in FLT3-ITD acute myeloid leukemia. Leukemia.

[97] Karginov FV, Cheloufi S, Chong MM, Stark A, Smith AD, Hannon GJ (2010). Diverse endonucleolytic cleavage sites in the mammalian transcriptome depend upon microRNAs, Drosha, and additional nucleases. Mol Cell.

[98] O'Carroll D, Mecklenbrauker I, Das PP, Santana A, Koenig U, Enright AJ (2007). A Slicer-independent role for Argonaute 2 in hematopoiesis and the microRNA pathway. Genes Dev.

[99] Iosue I, Quaranta R, Masciarelli S, Fontemaggi G, Batassa EM, Bertolami C (2013). Argonaute 2 sustains the gene expression program driving human monocytic differentiation of acute myeloid leukemia cells. Cell Death Dis.

[100] Gagnon KT, Corey DR (2012). Argonaute and the nuclear RNAs: new pathways for RNA-mediated control of gene expression. Nucleic Acid Ther.

[101] Daschkey S, Röttgers S, Giri A, Bradtke J, Teigler-Schlegel A, Meister G (2013). MicroRNAs distinguish cytogenetic subgroups in pediatric AML and contribute to complex regulatory networks in AML-relevant pathways. PLoS ONE.

[102] Jønson L, Christiansen J, Hansen Thomas VO, Vikeså J, Yamamoto Y, Nielsen FC (2014). IMP3 RNP safe houses prevent miRNA-directed HMGA2 mRNA decay in cancer and development. Cell Rep.

[103] Kundu P, Fabian MR, Sonenberg N, Bhattacharyya SN, Filipowicz W (2012). HuR protein attenuates miRNA-mediated repression by promoting miRISC dissociation from the target RNA. Nucleic Acids Res.

[104] Ennajdaoui H, Howard Jonathan M, Sterne-Weiler T, Jahanbani F, Coyne Doyle J, Uren Philip J (2016). IGF2BP3 modulates the interaction of invasion-associated transcripts with RISC. Cell Rep.

[105] Diaz-Muñoz MD, Bell SE, Fairfax K, Monzon-Casanova E, Cunningham AF, Gonzalez-Porta M (2015). The RNA-binding protein HuR is essential for the B cell antibody response. Nat Immunol.

[106] Ghosh M, Aguila HL, Michaud J, Ai Y, Wu M-T, Hemmes A (2009). Essential role of the RNA-binding protein HuR in progenitor cell survival in mice. J Clin Invest.

[107] Topisirovic I, Siddiqui N, Orolicki S, Skrabanek LA, Tremblay M, Hoang T (2009). Stability of eukaryotic translation initiation factor 4E mRNA is regulated by HuR, and this activity is dysregulated in cancer. Mol Cell Biol.

[108] Ishimaru D, Zuraw L, Ramalingam S, Sengupta TK, Bandyopadhyay S, Reuben A (2010). Mechanism of regulation of bcl-2 mRNA by nucleolin and A+U-rich element-binding factor 1 (AUF1). J Biol Chem.

[109] Ishimaru D, Ramalingam S, Sengupta TK, Bandyopadhyay S, Dellis S, Tholanikunnel BG (2009). Regulation of Bcl-2 expression by HuR in HL60 leukemia cells and A431 carcinoma cells. Mol Cancer Res.

[110] Kim HH, Kuwano Y, Srikantan S, Lee EK, Martindale JL, Gorospe M (2009). HuR recruits let-7/RISC to repress c-Myc expression. Genes Dev.

[111] Tominaga K, Srikantan S, Lee EK, Subaran SS, Martindale JL, Abdelmohsen K (2011). Competitive regulation of nucleolin expression by HuR and miR-494. Mol Cell Biol.

[112] Pickering BF, Yu D, Van Dyke MW (2011). Nucleolin protein interacts with microprocessor complex to affect biogenesis of microRNAs 15a and 16. J Biol Chem.

[113] Shen N, Yan F, Pang J, Wu L-C, Al-Kali A, Litzow MR (2014). A nucleolin-DNMT1 regulatory axis in acute myeloid leukemogenesis. Oncotarget.

[114] Chen M-T, Dong L, Zhang X-H, Yin X-L, Ning H-M, Shen C (2015). ZFP36L1 promotes monocyte/macrophage differentiation by repressing CDK6. Sci Rep.

[115] O'Connell RM, Rao DS, Chaudhuri AA, Baltimore D (2010). Physiological and pathological roles for microRNAs in the immune system. Nat Rev Immunol.

[116] Viswanathan SR, Powers JT, Einhorn W, Hoshida Y, Ng TL, Toffanin S (2009). Lin28 promotes transformation and is associated with advanced human malignancies. Nat Genet.

[117] Xu B, Zhang K, Huang Y (2009). Lin28 modulates cell growth and associates with a subset of cell cycle regulator mRNAs in mouse embryonic stem cells. RNA (New York, NY).

[118] Yuan J, Nguyen CK, Liu X, Kanellopoulou C, Muljo SA (2012). Lin28b reprograms adult bone marrow hematopoietic progenitors to mediate fetal-like lymphopoiesis. Science.

[119] Wang S, Chim B, Su Y, Khil P, Wong M, Wang X (2019). Enhancement of LIN28B-induced hematopoietic reprogramming by IGF2BP3. Genes Dev.

[120] Zhou J, Bi C, Ching YQ, Chooi J-Y, Lu X, Quah JY (2017). Inhibition of LIN28B impairs leukemia cell growth and metabolism in acute myeloid leukemia. J Hematol Oncol.

[121] Jiang X, Huang H, Li Z, Li Y, Wang X, Gurbuxani S (2012). Blockade of miR-150 maturation by MLL-fusion/MYC/LIN-28 is required for MLL-associated leukemia. Cancer Cell.

[122] Roos M, Pradère U, Ngondo RP, Behera A, Allegrini S, Civenni G (2016). A small-molecule inhibitor of Lin28. ACS Chem Biol.

[123] Okholm TLH, Sathe S, Park SS, Kamstrup AB, Rasmussen AM, Shankar A (2020). Transcriptome-wide profiles of circular RNA and RNA-binding protein interactions reveal effects on circular RNA biogenesis and cancer pathway expression. Genome Med.

[124] Salzman J, Gawad C, Wang PL, Lacayo N, Brown PO (2012). Circular RNAs are the predominant transcript isoform from hundreds of human genes in diverse cell types. PLoS ONE.

[125] Du WW, Yang W, Liu E, Yang Z, Dhaliwal P, Yang BB (2016). Foxo3 circular RNA retards cell cycle progression via forming ternary complexes with p21 and CDK2. Nucleic Acids Res.

[126] Hansen TB, Jensen TI, Clausen BH, Bramsen JB, Finsen B, Damgaard CK (2013). Natural RNA circles function as efficient microRNA sponges. Nature.

[127] Zhang Y, Zhang XO, Chen T, Xiang JF, Yin QF, Xing YH (2013). Circular intronic long noncoding RNAs. Mol Cell.

[128] Schneider T, Hung L-H, Schreiner S, Starke S, Eckhof H, Rossbach O (2016). CircRNA-protein complexes: IMP3 protein component defines subfamily of circRNPs. Sci Rep.

[129] Ashwal-Fluss R, Meyer M, Pamudurti NR, Ivanov A, Bartok O, Hanan M (2014). circRNA biogenesis competes with pre-mRNA splicing. Mol Cell.

[130] Conn SJ, Pillman KA, Toubia J, Conn VM, Salmanidis M, Phillips CA (2015). The RNA binding protein quaking regulates formation of circRNAs. Cell.

[131] Jeck WR, Sorrentino JA, Wang K, Slevin MK, Burd CE, Liu J (2013). Circular RNAs are abundant, conserved, and associated with ALU repeats. RNA.

[132] Guarnerio J, Bezzi M, Jeong Jong C, Paffenholz Stella V, Berry K, Naldini Matteo M (2016). Oncogenic role of fusion-circRNAs derived from cancer-associated chromosomal translocations. Cell.

[133] Huang W, Fang K, Chen TQ, Zeng ZC, Sun YM, Han C (2019). circRNA circAF4 functions as an oncogene to regulate MLL-AF4 fusion protein expression and inhibit MLL leukemia progression. J Hematol Oncol.

[134] Sun Y-M, Wang W-T, Zeng Z-C, Chen T-Q, Han C, Pan Q (2019). circMYBL2, a circRNA from MYBL2, regulates FLT3 translation by recruiting PTBP1 to promote FLT3-ITD AML progression. Blood.

[135] Kharas MG, Lengner CJ, Al-Shahrour F, Bullinger L, Ball B, Zaidi S (2010). Musashi-2 regulates normal hematopoiesis and promotes aggressive myeloid leukemia. Nat Med.

[136] Byers RJ, Currie T, Tholouli E, Rodig SJ, Kutok JL (2011). MSI2 protein expression predicts unfavorable outcome in acute myeloid leukemia. Blood.

[137] Park S-M, Gönen M, Vu L, Minuesa G, Tivnan P, Barlowe TS (2015). Musashi2 sustains the mixed-lineage leukemia-driven stem cell regulatory program. J Clin Invest.

[138] Vu LP, Prieto C, Amin EM, Chhangawala S, Krivtsov A, Calvo-Vidal MN (2017). Functional screen of MSI2 interactors identifies an essential role for SYNCRIP in myeloid leukemia stem cells. Nat Genet.

[139] Minuesa G, Albanese SK, Xie W, Kazansky Y, Worroll D, Chow A (2019). Small-molecule targeting of MUSASHI RNA-binding activity in acute myeloid leukemia. Nat Commun.

[140] Bell JL, Wächter K, Mühleck B, Pazaitis N, Köhn M, Lederer M (2013). Insulin-like growth factor 2 mRNA-binding proteins (IGF2BPs): post-transcriptional drivers of cancer progression?. Cell Mol Life Sci.

[141] Huang H, Weng H, Sun W, Qin X, Shi H, Wu H (2018). Recognition of RNA N6-methyladenosine by IGF2BP proteins enhances mRNA stability and translation. Nat Cell Biol.

[142] Stöhr N, Lederer M, Reinke C, Meyer S, Hatzfeld M, Singer RH (2006). ZBP1 regulates mRNA stability during cellular stress. J Cell Biol.

[143] Palanichamy JK, Tran TM, Howard JM, Contreras JR, Fernando TR, Sterne-Weiler T (2016). RNA-binding protein IGF2BP3 targeting of oncogenic transcripts promotes hematopoietic progenitor proliferation. J Clin Invest.

[144] Stoskus M, Gineikiene E, Valceckiene V, Valatkaite B, Pileckyte R, Griskevicius L (2011). Identification of characteristic IGF2BP expression patterns in distinct B-ALL entities. Blood Cells Mol Dis.

[145] Andersson A, Olofsson T, Lindgren D, Nilsson B, Ritz C, Edén P (2005). Molecular signatures in childhood acute leukemia and their correlations to expression patterns in normal hematopoietic subpopulations. Proc Natl Acad Sci USA.

[146] King RL, Pasha T, Roullet MR, Zhang PJ, Bagg A (2009). IMP-3 is differentially expressed in normal and neoplastic lymphoid tissue. Hum Pathol.

[147] Wilkinson KA, Merino EJ, Weeks KM (2006). Selective 2'-hydroxyl acylation analyzed by primer extension (SHAPE): quantitative RNA structure analysis at single nucleotide resolution. Nat Protoc.

[148] Dargyte M, Philipp J, Palka CD, Stone MD, Sanford JR. Splicing factor SRSF1 expands the regulatory logic of microRNA expression. bioRxiv. 2020:2020.05.12.092270.

[149] Liu H, Begik O, Lucas MC, Ramirez JM, Mason CE, Wiener D (2019). Accurate detection of m6A RNA modifications in native RNA sequences. Nat Commun.

